# Climate anxiety, pro-environmental behavior, and perceived barriers in university students: analysis of a publicly available dataset from the United States

**DOI:** 10.3389/fpsyg.2026.1889006

**Published:** 2026-07-27

**Authors:** Fanni Küplen, Angéla Somogyi, Robert Podstawski, Celal Bulgay, Attila Szabo

**Affiliations:** 1 Department of Psychology and Health Management, Faculty of Health and Sport Sciences, Széchenyi István University, Győr, Hungary; 2 Department of Physiotherapy, University of Warmia and Mazury in Olsztyn, Olsztyn, Poland; 3 Faculty of Sport Sciences, Bingöl University, Bingöl, Türkiye

**Keywords:** Climate Anxiety Scale, climate change anxiety, perceived barriers, Poisson regression, pro-environmental behavior, university students

## Abstract

**Introduction:**

Climate change anxiety is increasingly recognized as an important psychological response to the climate crisis among young adults, but its link to climate-related behaviors remains unclear.

**Methods:**

Using the four-factor Climate Anxiety Scale (CAS), this secondary analysis of a public dataset examined whether cognitive-emotional difficulties, functional impairment, personal experience with climate change, and behavioral engagement predicted pro-environmental behavior (PEB) and perceived barriers to climate action in a sample of American university students (*N* = 435; ages 18–22; 72.6% female). Composite scores derived from five PEB categories and five barrier categories were analyzed with Poisson regression, complemented by binomial sensitivity analyses.

**Results:**

Both Poisson models were significant [PEB: LR *χ*^2^(4) = 62.57, *p* < 0.001; perceived barriers: LR *χ*^2^(4) = 46.32, *p* < 0.001], and behavioral engagement was the only significant predictor in each (IRRs = 1.049 and 1.035, respectively). In sex-stratified models, behavioral engagement predicted PEB among females, whereas personal experience predicted PEB among males; behavioral engagement predicted perceived barriers in both groups. Binomial sensitivity analyses largely confirmed these patterns, with behavioral engagement the most consistent predictor across outcomes, sexes, and analytic approaches.

**Discussion:**

Together, these findings suggest that it is the behavioral and motivational components of the CAS, rather than climate anxiety as a global construct, that are most consistently associated with PEB and barrier awareness. By contrast, the distress-related dimensions showed little independent association with either outcome apart from a small model-dependent association between Cognitive–Emotional Difficulties and PEB in the binomial sensitivity analyses. These findings underscore the value of treating climate anxiety as a multidimensional construct in research and intervention efforts aimed at translating climate concern into sustained action.

## Introduction

Climate change represents one of the defining challenges of the twenty-first century, with wide-ranging consequences for ecosystems, human health, and social stability (Masson-Delmotte et al., 2018). Beyond its direct physical impacts, climate change is increasingly recognized as a significant source of psychological distress (Clayton, 2020; Doherty and Clayton, 2011). A growing body of research has documented that many individuals, particularly young adults, experience persistent worry, rumination, and functional difficulties related to climate change (Clayton and Karazsia, 2020; Stanley et al., 2021). These emotional responses have been collectively described under the umbrella of climate anxiety (also termed eco-anxiety), which refers to a spectrum of negative affective reactions to perceived threats posed by anthropogenic climate change (Coffey et al., 2021; Pihkala, 2020). Researchers have increasingly emphasized that climate anxiety is only one component of a broader spectrum of “eco-emotions,” which may include eco-guilt, eco-grief, anger, and feelings of helplessness (Coffey et al., 2021).

Despite growing scientific and public attention to climate anxiety, important questions remain regarding its psychological structure and its relationship to behavioral outcomes. More specifically, whether and how climate anxiety translates into pro-environmental behavior (PEB) or action, or alternatively, into perceived helplessness and inhibition, has been the subject of considerable theoretical debate (Doherty and Clayton, 2011; Verplanken et al., 2020). Some scholars believe that climate anxiety can serve an adaptive function, motivating individuals to engage in protective PEBs (Kühner et al., 2024; Stanley et al., 2021). Others have argued that high levels of anxiety may produce cognitive and functional impairments that weaken effective engagement with the problem (Clayton and Karazsia, 2020; Verplanken et al., 2020). Resolving this tension requires an empirical examination of how specific dimensions of climate anxiety relate differentially to climate-relevant behavior. To address these questions, it is necessary to examine climate anxiety using measurement tools that capture its multidimensional structure. Critically, the targeted central contribution of the present work is not simply that climate anxiety, as a global construct, relates to behavior, but that climate anxiety should possibly be treated as a multidimensional construct whose distress-related dimensions (i.e., cognitive-emotional difficulties and functional impairment) and behavioral or motivational engagement dimension may relate notably differently to climate-relevant outcomes. Clarifying this distinction, rather than testing climate anxiety as a unitary construct, is the specific gap addressed by the present study.

### The Climate Anxiety Scale and its factor structure

Clayton and Karazsia (2020) developed the first validated self-report instrument for assessing climate anxiety: the 22-item Climate Anxiety Scale (CAS). The CAS was designed to capture the multidimensional nature of the construct and was validated via exploratory and confirmatory factor analyses in U.S. samples, yielding a four-factor structure. The first factor, cognitive-emotional difficulties, includes rumination, nightmares, crying, and poor concentration in response to climate change (items 1–8). The second factor, functional impairment, captures the extent to which climate anxiety disrupts the person’s ability to engage in work, social interactions, and daily activities (items 9–13). The third factor, personal experience of climate change, reflects direct and indirect exposure to climate-related events (items 14–16). The fourth factor, behavioral engagement, reflects pro-environmental motivation and adaptive behavioral responses, including recycling, reducing energy use, and guilt about wasteful behavior (items 17–22).

Subsequent validation studies have refined the understanding of the CAS’s psychometric properties. Wullenkord et al. (2021) examined a German 12-item adaptation and found that the two-factor structure (cognitive-emotional difficulties and functional impairment) provided better fit than a unidimensional model, though fit indices were suboptimal. Mouguiama-Daouda et al. (2022) conducted two preregistered confirmatory factor analytic studies with French-speaking community samples (*n* = 305 and *n* = 905) and found consistent support for a two-factor structure comprising the first 13 items—covering cognitive-emotional difficulties and functional impairment—as superior to both the full four-factor model and a unidimensional alternative. Critically, Mouguiama-Daouda et al. (2022) observed that both factors were positively associated with depression and environmental identity, but not with generalized anxiety, raising important questions about the discriminant validity of the CAS vis-à-vis established anxiety measures. Across these studies, the behavioral engagement and personal experience subscales have been treated as theoretically distinct from the core anxiety components and have generally shown weak associations with the distress-laden subscales.

### Climate anxiety in young adults

University students have emerged as a particularly salient population for the study of climate anxiety. This developmental stage, often referred to as emerging adulthood, is characterized by identity exploration, increasing autonomy, and the consolidation of long-term behavioral patterns that may persist into later adulthood (Arnett et al., 2014). Large international surveys indicate that climate-related worry is especially prevalent among younger generations. Hickman et al. (2021) surveyed over 10,000 young people aged 16–25 across ten countries and found that 59% reported feeling very or extremely worried about climate change, while nearly half stated that these concerns negatively affected their daily functioning. Young adults aged 18 to 25 also exhibit heightened climate-related worry compared with older groups, likely due to a combination of developmental factors, greater perceived future exposure to climate impacts, and evolving political and social identities (Clayton, 2020; Ojala et al., 2021). College campuses represent environments in which climate change is both an academic topic and a socially salient concern, making university students an important group for understanding the psychological and behavioral dimensions of climate anxiety. Moreover, behavioral patterns established during emerging adulthood—including pro-environmental habits, political engagement, and consumption choices—may have long-term implications for societal climate outcomes.

### Pro-environmental behavior and perceived barriers

Pro-environmental behavior refers to actions that minimize negative environmental impacts or actively benefit the environment, including sustainable consumption, reduced energy and water use, sustainable transportation, dietary change, and climate advocacy (Steg and Vlek, 2009). Despite research consistently identifying environmental values, attitudes, and concern as predictors of PEB (Bamberg and Möser, 2007), the relationship between anxiety and action is more nuanced. Anxiety may mobilize action, or coping behavior, but it may also trigger avoidance when the perceived threat is overwhelming, and self-efficacy is low (Verplanken et al., 2020). The distinction between constructive and unconstructive worry articulated by Verplanken et al. (2020) is especially relevant here: individuals with habitual eco-anxiety may be less, rather than more, likely to act.

Alongside behavioral outcomes, perceptions of barriers to climate action are an important psychological variable that shapes engagement with the climate problem (Lorenzoni et al., 2007). Perceived barriers, including financial constraints, lack of institutional support, social or cultural resistance, and limited technological options, can inhibit individual action, even among those concerned about climate change. Research on barrier perception suggests that individuals who are more engaged with climate change at a motivational level may simultaneously be more aware of the structural obstacles that impede meaningful action (Lorenzoni et al., 2007). Whether climate anxiety, particularly its behavioral engagement component, predicts heightened awareness of such barriers remains underexplored.

### The present study

The present study was a secondary analysis of an existing dataset examining the relationships between the four dimensions of climate anxiety, as measured by the CAS, and two climate-relevant outcomes—PEB and perceived barriers to climate action—in a sample of university students. Both outcomes were operationalized as composite counts derived from binary endorsements of specific action and barrier categories, making Poisson regression the appropriate analytic framework. Drawing on the distinction between the distress-related components of climate anxiety (cognitive-emotional difficulties and functional impairment) and its motivational component (behavioral engagement), we expected behavioral engagement to positively predict both PEB and barrier perception. The roles of cognitive-emotional difficulties and functional impairment were examined exploratorily, given prior theory supporting both anxiety-facilitation and anxiety-inhibition hypotheses.

## Method

### Data source

Data and associated metadata used in this study were obtained from the Mendeley Data repository (Perttula, 2026; https://doi.org/10.17632/6bc2mbztmy.1). The open-access dataset was used in accordance with the Creative Commons Attribution 4.0 International license (CC BY 4.0; https://creativecommons.org/licenses/by/4.0/). *Use of these materials does not imply consent from or collaboration with the original author (i.e., Perttula)*. According to the accompanying SPSS data file, data collection occurred between September 30, 2024, and December 6, 2024. The variable ‘CSUStatus,’ which asks respondents whether they attend Colorado State University, indicates that 450 of the 452 respondents answered yes (coded as 1), with 2 observations missing.

The dataset included only age-group categories: respondents aged 18–22 (*n* = 435) were coded as 2, and those aged 23–100 (*n* = 16) were coded as 3. A category coded as 1 was intended to represent those aged 15–17, but no observations were recorded for this group. One additional response was missing. To focus on a homogeneous group of young adults, only responses coded as 2 (96.2% of the data) were retained, resulting in 17 exclusions (16 aged 23 or older and one with missing age), which is the sole modification of the original (*n* = 452–17 = 435) dataset.

This restriction was applied because the 18–22 age band captures a developmentally coherent period of emerging adulthood (Arnett et al., 2014) and constituted the vast majority of the sample (96.2%), whereas the 23–100 category was both small (*n* = 16) and internally heterogeneous, spanning a much wider and less developmentally uniform age range. Restricting the sample in this way increases the internal comparability of participants but narrows the generalizability of the findings: individuals aged 23 and older, who may differ in life circumstances (e.g., employment, family responsibilities, further education) and in the duration or nature of their exposure to climate-related information, are not represented, and the present results should not be extended to non-traditional or older university students without further study.

### Ethics and procedure

This study was a secondary analysis of an open-access dataset published by Perttula (2026) under the CC BY 4.0 license. The set of documents in the publicly available dataset indicates that the original study protocol was reviewed by an Institutional Review Board at the time of data collection and was determined to be exempt under Category 2 of the U.S. Common Rule (45 CFR 46.104(d)(2)). Participation in the original study was voluntary and anonymous, and written informed consent was obtained from all participants. Based on the file structure and documentation, the data were collected via an online self-report survey administered through Qualtrics. The original study was administered to volunteers aged 18 years and older on the Qualtrics platform in a fully anonymous manner, with data collection occurring between September 30 and December 6, 2024 (Perttula, 2026). According to the recruitment materials accompanying the dataset, participants were invited to take part because they were at least 18 years old and were enrolled in a course in the Psychology Department at Colorado State University that participates in the department’s research pool; the stated purpose communicated to participants was to examine “preferences and experiences as they relate to personality, health risk behaviors, and how these are related to thoughts about climate change.” Beyond this description, the publicly available documentation does not specify further details of the sampling strategy (e.g., whether participation was tied to course credit, open announcements, or another mechanism) or the exact location(s) where recruitment occurred, and no information on the size of the eligible pool or the response rate is provided. Because the present study is a secondary analysis, the authors had no control over the original sampling procedure, and this lack of detail is addressed as a limitation later.

Although no additional ethical approval was required for the present secondary analysis of publicly available anonymized data, we nevertheless requested and obtained permission to use the dataset from the Science Ethics Committee of the Scientific Advisory Board of Széchenyi István University (Permission No. SZE/ETT-39/2026 [III.17.]). For the present hypothesis testing, we used demographic variables, Climate Anxiety Scale (CAS) scores, five pro-environmental behaviors (PEBs), and five perceived barriers to PEB from the dataset.

### Participants

Participants included 435 CSU students. The sample was primarily female (*n* = 316, 72.6%), with 118 males (27.1%) and one participant who identified as intersex (0.2%). All participants were between 18 and 22 years old. Racial and ethnic backgrounds were mainly White (*n* = 317, 72.9%), followed by Multiracial (n = 59, 13.6%), Latina/o (*n* = 30, 6.9%), Asian (*n* = 12, 2.8%), Black (*n* = 9, 2.1%), Indigenous (*n* = 4, 0.9%), Middle Eastern (*n* = 3, 0.7%), and one person who preferred not to disclose their race (0.2%). Regarding sexual orientation, 71.7% identified as heterosexual or straight, 15.6% as bisexual, 4.6% as queer, 3.7% as gay or lesbian, 2.8% as questioning or unsure, 0.2% as other, and 0.7% preferred not to answer.

### Measures

#### Climate Anxiety Scale

The CAS (Clayton and Karazsia, 2020) is a 22-item self-report measure assessing anxiety related to climate change across four subscales. Items are rated on a 5-point Likert scale from 1 (Never) to 5 (Almost Always). Subscale scores are computed as the sum of items within each subscale. The four subscales are: (a) Cognitive-Emotional Difficulties (items 1–8; e.g., “Thinking about climate change makes it difficult for me to concentrate”; theoretical range 8–40), reflecting rumination and emotional dysregulation in response to climate change (Cronbach’s *α* = 0.88 in the present dataset); (b) Functional Impairment (items 9–13; e.g., “My concern about climate change interferes with my ability to get work or school assignments done”; theoretical range 5–25), capturing disruptions to daily functioning (*α* = 0.88); (c) Personal Experience of Climate Change (items 14–16; e.g., “I have been directly affected by climate change”; theoretical range 3–15), reflecting direct and vicarious exposure (*α* = 0.88); and (d) Behavioral Engagement (items 17–22; e.g., “I try to reduce my behaviors that contribute to climate change”; theoretical range 6–30), indexing adaptive, pro-environmental motivation (*α* = 0.82). Observed score ranges and means are reported in Table 1. CAS subscale scores were calculated as raw item sums only for cases with complete data on the constituent items of each subscale.

**Table 1:** Descriptive statistics for study variables.

Variable	*n*	*M*	SD	Min	Max
CAS subscales (predictors)					
Cognitive-emotional difficulties	432	10.85	4.10	8	26
Functional impairment	434	5.90	2.17	5	21
Personal experience	434	5.99	3.32	3	15
Behavioral engagement	434	19.63	4.99	6	30
Outcome variables					
Pro-environmental behavior	435	1.92	1.23	0	5
Perceived barriers	435	2.58	1.29	0	5

CAS, Climate Anxiety Scale (Clayton and Karazsia, 2020). CAS subscale scores are raw item sums calculated only for cases with complete data on the constituent items; therefore, the n differs across CAS subscales. Outcome variables are composite count scores that reflect the number of endorsed categories (range: 0–5).

#### Pro-environmental behavior composite

Pro-environmental behavior was assessed by asking participants to indicate (yes/no) which actions they had taken to reduce their carbon footprint from a list of five categories: (1) energy and water efficiency (e.g., energy-efficient appliances, reduced water use, solar panels); (2) sustainable transportation (e.g., public transit, carpooling, cycling); (3) sustainable consumption and waste reduction (e.g., locally sourced or organic food); (4) diet and lifestyle changes (e.g., plant-based or reduced-meat diet); and (5) engagement in climate activism or advocacy (e.g., joining pro-environmental groups or attending protests). Responses were summed to create a composite count score of the *yes* answers (range 0–5) reflecting the number of distinct action categories endorsed.

#### Perceived barriers composite

Perceived barriers to addressing climate change were assessed by asking participants to indicate (yes/no) which factors they perceived as major barriers from a list of five categories: (1) lack of awareness; (2) financial constraints; (3) lack of governmental or institutional support; (4) technological limitations; and (5) social or cultural resistance. As with PEB, responses were summed to create a composite *yes*-count score (range 0–5).

#### Data analysis strategy

Two separate Poisson regression models were estimated within the generalized linear model (GLM) framework using a log link function, with PEB and barriers specified as the respective outcome variables and the four CAS subscale scores entered simultaneously as predictors in each model. Both dependent variables were bounded count measures (0–5), making Poisson regression an appropriate modeling approach. Poisson regression models the natural logarithm of the expected count as a linear function of the predictors (Cameron and Trivedi, 2013). Regression coefficients (B) are estimated on the log scale, and incidence rate ratios (IRRs; exp.[B]) are reported to facilitate interpretation. For example, an IRR of 1.05 indicates a 5% increase in the expected count for each one-unit increase in the predictor. It should be noted that although PEB and perceived barriers are bounded counts (0–5) rather than unbounded event counts in the strict sense, and are constructed as sums of five binary indicators, Poisson regression remains a common and interpretable modeling choice for such composite scores because it directly models expected counts and yields readily interpretable IRRs (Cameron and Trivedi, 2013). We nonetheless recognize that binomial, quasibinomial, ordinal, or robust-standard-error models represent viable alternative specifications for bounded sum scores of this kind, and we present dispersion diagnostics (Pearson *χ*^2^/df and Deviance/df) to evaluate whether the Poisson variance assumption was reasonably met. Because both outcomes were bounded sums of five binary indicators, binomial sensitivity analyses were also conducted, treating each outcome as the number of endorsed categories out of five. These analyses were used to examine whether the substantive interpretation of the Poisson models was robust to an alternative specification more directly aligned with the summed-binary structure of the outcomes. The results of these sensitivity analyses are reported in Supplementary material.

Model fit was evaluated using the omnibus likelihood ratio (LR) chi-square test and dispersion diagnostics (Pearson χ^2^/df and Deviance/df), where values close to 1.0 indicate appropriate dispersion. Dispersion statistics were examined to assess potential overdispersion. Statistical significance of individual parameters was evaluated using Wald chi-square tests. The significance threshold was set at *α* = 0.05, and 95% Wald confidence intervals are reported for all estimates. All analyses were conducted in IBM SPSS Statistics using the GENLIN procedure with Fisher scoring estimation.

## Results

### Descriptive statistics

Descriptive statistics for all study variables are presented in Table 1. Pro-environmental behavior scores ranged from 0 to 5 (*M* = 1.92, SD = 1.23), with participants endorsing an average of approximately two distinct action categories. Table 1 also presents descriptive statistics for the four CAS subscales used as predictors in the regression models. The most frequently endorsed actions were sustainable transportation and energy and water efficiency, followed by sustainable consumption, diet and lifestyle changes, and climate activism or advocacy. Perceived barriers ranged from 0 to 5 (*M* = 2.58, SD = 1.29). The most frequently endorsed barriers were lack of awareness and lack of governmental or institutional support, followed by financial constraints, social or cultural resistance, and technological limitations. Table 2 reports the percentage of participants endorsing each of the five perceived-barrier categories individually, along with the corresponding percentages for the PEB categories summarized above.

**Table 2:** Endorsement frequencies for pro-environmental behavior and perceived barrier categories.

Category (PEB)	% Endorsing (PEB)	Category (barriers)	% Endorsing (barrier)
Energy and water efficiency	53.6%	Lack of awareness	72.2%
Sustainable transportation	57.0%	Financial constraints	48.7%
Sustainable consumption/waste reduction	43.9%	Lack of governmental or institutional support	67.4%
Diet and lifestyle changes	25.7%	Technological limitations	23.0%
Climate activism or advocacy	12.2%	Social or cultural resistance	47.1%

PEB, pro-environmental behavior; percentages reflect the proportion of participants (*N* = 435) endorsing each category.

Among the CAS subscales, behavioral engagement yielded the highest mean score (*M* = 19.63, SD = 4.99; range 6–30), consistent with a sample that was generally motivated toward climate-relevant action even if not clinically anxious. Cognitive–emotional difficulties scores were low on average (*M* = 10.85, SD = 4.10; range 8–26), as were functional impairment scores (*M* = 5.90, SD = 2.17; range 5–21), indicating that marked disruption of daily functioning due to climate anxiety was uncommon in this non-clinical sample. Personal experience scores were also modest (*M* = 5.99, SD = 3.32; range 3–15).

### Poisson regression

Two Poisson regression models were estimated to examine the association between the four CAS subscales (cognitive–emotional difficulties, functional impairment, personal experience, and behavioral engagement) and two outcomes: PEB and perceived barriers to PEB. Both models demonstrated adequate dispersion and were statistically significant according to likelihood ratio tests. Across both models, behavioral engagement emerged as the only significant predictor, whereas cognitive–emotional difficulties, functional impairment, and personal experience were not significantly associated with either outcome. Full model estimates are presented in Tables 3, 4. Binomial sensitivity analyses, treating each outcome as the number of endorsed categories out of five, broadly supported the Poisson results. Behavioral Engagement remained a significant predictor of both outcomes. However, Cognitive–Emotional Difficulties also emerged as a significant predictor of Pro-Environmental Behavior in the binomial model. Full sensitivity results are reported in Supplementary Tables S1–S4.

**Table 3:** Poisson regression predicting pro-environmental behavior.

Predictor	*B*	*SE*	*IRR*	*95% CI*	*p*
Cognitive–emotional difficulties	0.020	0.013	1.020	0.995–1.046	0.121
Functional impairment	−0.021	0.022	0.979	0.938–1.023	0.345
Personal experience	0.009	0.013	1.009	0.985–1.035	0.465
Behavioral engagement	0.048	0.009	1.049	1.030–1.068	< 0.001

Model statistics: LR *χ*^2^(4) = 62.57, *p* < 0.001. IRR, incidence rate ratio. The analytic sample size is N = 430 due to five incomplete answers on the CAS.

**Table 4:** Poisson regression predicting perceived barriers.

Predictor	*B*	*SE*	*IRR*	*95% CI*	*p*
Cognitive–emotional difficulties	0.010	0.011	1.010	0.987–1.032	0.400
Functional impairment	−0.010	0.019	0.990	0.953–1.029	0.618
Personal experience	0.013	0.011	1.013	0.991–1.035	0.250
Behavioral engagement	0.034	0.008	1.035	1.019–1.051	< 0.001

Model statistics: LR *χ*^2^(4) = 46.32, *p* < 0.001. IRR, incidence rate ratio. The analytic sample size is *N* = 430 due to five incomplete answers on the CAS.

### Sex-stratified Poisson regression predicting pro-environmental behavior

The sex-stratified models were estimated separately for female (*n* = 311) and male (*n* = 118) participants; the one participant who identified as intersex was excluded from these models. Sex-stratified Poisson regression models were estimated to examine whether the four CAS subscales predicted PEB separately for female and male participants. Both models showed adequate fit and no evidence of overdispersion. The overall model was significant in both groups, but the pattern of significant predictors differed by sex: among females, behavioral engagement was the only significant predictor of PEB, whereas among males, personal experience was the only significant predictor. Behavioral engagement showed a positive but non-significant trend in males. Full coefficients are presented in Table 5. In the corresponding binomial sensitivity analyses, Cognitive–Emotional Difficulties also emerged as a significant predictor of Pro-Environmental Behavior among females, while Behavioral Engagement became significant among males alongside Personal Experience. Full sensitivity results are reported in Supplementary Tables S1–S4.

**Table 5:** Poisson regression predicting pro-environmental behavior by sex.

Predictor	*♀ B*	*SE*	*IRR*	*95% CI*	*p*	*♂ B*	*SE*	*IRR*	*95% CI*	*p*
Cognitive–emotional difficulties	0.024	0.014	1.025	0.997–1.052	0.077	0.000	0.036	1.000	0.932–1.073	0.999
Functional impairment	−0.011	0.023	0.989	0.947–1.036	0.642	−0.041	0.065	0.960	0.845–1.090	0.529
Personal experience	−0.007	0.015	0.993	0.965–1.021	0.612	0.062	0.025	1.064	1.013–1.117	0.014
Behavioral engagement	0.046	0.010	1.047	1.026–1.068	< 0.001	0.039	0.021	1.040	0.998–1.083	0.065

Female model (♀): LR *χ*^2^(4) = 38.09, *p* < 0.001; Deviance/df = 0.691; Pearson *χ*^2^/df = 0.656; *n* = 311. Male model (**♂**): LR *χ*^2^(4) = 20.94, *p* < 0.001; Deviance/df = 0.919; Pearson *χ*^2^/df = 0.762; *n* = 118. IRR, incidence rate ratio. Confidence intervals for IRRs were obtained by exponentiating the reported 95% Wald confidence limits for B.

### Sex-stratified Poisson regression predicting perceived barriers

Sex-stratified Poisson regression models were estimated to examine whether the four CAS subscales predicted perceived barriers separately for female and male participants. Both models showed adequate fit and no evidence of overdispersion. The overall model was significant in both groups, and the pattern of predictors was similar across sex: in both females and males, behavioral engagement was the only significant predictor of perceived barriers, whereas cognitive-emotional difficulties, functional impairment, and personal experience were not significantly associated with the outcome. Full coefficients are presented in Table 6. For perceived barriers, the binomial sensitivity analyses confirmed Behavioral Engagement as a significant predictor in both females and males, with no additional CAS subscale reaching statistical significance. Full sensitivity results are reported in Supplementary Tables S1–S4.

**Table 6:** Poisson regression predicting perceived barriers by sex.

Predictor	*♀ B*	*SE*	*IRR*	*95% CI*	*p*	*♂ B*	*SE*	*IRR*	*95% CI*	*p*
Cognitive–emotional difficulties	0.005	0.012	1.005	0.981–1.030	0.659	0.007	0.033	1.007	0.944–1.075	0.829
Functional impairment	−0.009	0.021	0.991	0.951–1.033	0.673	0.014	0.056	1.014	0.908–1.132	0.804
Personal experience	0.014	0.013	1.014	0.989–1.039	0.278	0.018	0.024	1.018	0.972–1.066	0.436
Behavioral engagement	0.027	0.009	1.028	1.010–1.046	0.002	0.036	0.018	1.037	1.002–1.074	0.039

Female model (♀): LR *χ*^2^(4) = 21.84, *p* < 0.001; Deviance/df = 0.545; Pearson *χ*^2^/df = 0.519; *n* = 311. Male model (♂): LR *χ*^2^(4) = 16.19, *p* = 0.003; Deviance/df = 0.753; Pearson *χ*^2^/df = 0.708; *n* = 118. IRR, incidence rate ratio. Confidence intervals for IRRs were obtained by exponentiating the 95% Wald confidence limits for B.

## Discussion

This study examined the relationships between the four dimensions of the Climate Anxiety Scale and two climate-relevant outcomes—PEB and perceived barriers to climate action—among university students. In the primary Poisson regression models, a consistent, theoretically informative pattern emerged: of the four CAS subscales, only behavioral engagement significantly predicted both outcomes, whereas the distress-related components—cognitive-emotional difficulties and functional impairment—were not significant predictors. These findings contribute to an emerging literature on the differential consequences of distinct dimensions of climate anxiety and have implications for theory, measurement, and intervention.

The binomial sensitivity analyses generally supported this interpretation, particularly the central role of behavioral engagement. However, they also indicated that Cognitive-Emotional Difficulties were positively associated with Pro-Environmental Behavior when the outcome was modeled as the number of endorsed categories out of five. This additional finding suggests that distress-related climate concern may have a modest association with behavioral endorsement under a model specification that is more directly aligned with the outcome’s summed-binary structure. Nevertheless, this effect was not observed in the primary Poisson model and did not consistently extend across both outcomes; therefore, it should be interpreted with caution and treated as a sensitivity finding rather than the main result.

### Behavioral engagement as the active ingredient

The most striking finding of the present study is the consistent and exclusive predictive role of behavioral engagement across both outcomes. Individuals who scored higher on the behavioral engagement subscale—reflecting motivated orientation toward pro-environmental action, recycling, guilt about energy waste, and a sense of personal agency—endorsed more pro-environmental action categories (IRR = 1.049) and perceived a greater number of barriers to climate action (IRR = 1.035). This double role of behavioral engagement is theoretically coherent. On the one hand, the behavioral engagement subscale of the CAS assesses constructs closely aligned with established predictors of PEB, including environmental motivation, personal norm activation, and a sense of individual responsibility (Steg and Vlek, 2009). Individuals higher on this dimension are, by definition, more oriented toward climate-protective action and thus more likely to report having taken it. On the other hand, the positive association between behavioral engagement and perceived barriers likely reflects the cognitive accessibility of obstacles among those actively attempting to engage with the problem. Individuals who are more engaged with climate change at a motivational level may simultaneously be more aware of the structural obstacles that impede meaningful action—a pattern consistent with research documenting greater barrier recognition among more engaged individuals (Lorenzoni et al., 2007). This interpretation is also consistent with broader sustainability research showing that environmental action is constrained not only by individual motivation but also by dominant social, institutional, and value systems that determine which relationships with nature are recognized and acted upon (Pascual et al., 2023).

These results align with the conceptual distinction made by Verplanken et al. (2020) between constructive and unconstructive eco-anxiety. In their view, constructive worry focuses on problem-solving and behavior change, whereas unconstructive worry involves rumination and avoidance. The behavioral engagement subscale of the CAS appears to measure the constructive end of this spectrum: it predicts action without the inhibitory effects typically associated with distress-related anxiety. This interpretation is also consistent with Stanley et al.’s (2021) finding that eco-anxiety can differentially predict adaptive coping depending on its emotional quality: anger and engaged concern were associated with action, whereas paralytic fear was not.

The present findings are also consistent with work on environmental cognitive alternatives and collective action. Wright et al. (2021) showed that individuals who could more readily imagine sustainable alternatives to the environmental status quo reported stronger politicized environmental identity, greater willingness to engage in pro-environmental activism, and were more likely to complete an observed activist behavior. Although our study did not measure cognitive alternatives or politicized environmental identity, the results point in a compatible direction. For example, climate-related action was predicted not by distress-related CAS components, but by behavioral engagement, a motivational and action-oriented dimension. This suggests that pro-environmental behavior may depend less on climate anxiety per se and more on whether concern is accompanied by perceived agency, imagined alternatives, and behavioral pathways for action.

In line with the recent call for greater transparency in PEB measurement (Kleinhansl et al., 2026), we document behavioral indices that encompass the PEB outcome. However, the measure should be interpreted as a count of selected self-reported climate-relevant action categories rather than as a comprehensive or normative operationalization of PEB. Although the five indicators capture breadth of engagement across several domains, they do not assess behavioral frequency, intensity, or environmental impact (Gatersleben et al., 2002). Moreover, because PEB indicators are context-specific and may change over time in response to regional policy, technology, and social norms, future research should situate these indicators within broader PEB frameworks and, where possible, use validated instruments.

### Sex differences in predictive patterns

Sex-stratified analyses revealed that the action-oriented component of climate anxiety operates somewhat differently across sex, particularly for pro-environmental behavior. Among women, behavioral engagement was the only significant predictor of PEB, consistent with evidence that more constructive, action-focused forms of eco-anxiety are positively associated with climate action rather than paralysis (Stanley et al., 2021; Verplanken et al., 2020). Among men, by contrast, personal experience was the only significant predictor of PEB, suggesting that direct or indirect lived experience of climate impacts may be a stronger pathway to action in this subgroup than generalized motivational engagement alone; this interpretation aligns with research showing that personal experience can heighten climate risk salience and strengthen behavioral responses under certain conditions, particularly when structural barriers and efficacy beliefs are simultaneously favorable (van der Linden, 2014), which may explain why this effect emerged only in men and not in the full sample or among women.

### Distress-related components: primary and sensitivity findings

In the primary Poisson models, the absence of significant effects for cognitive-emotional difficulties and functional impairment is equally noteworthy. Although both subscales showed numerically small positive associations with PEB, neither reached statistical significance, and confidence intervals were narrow and centered on zero. This null result challenges both the anxiety-facilitation hypothesis (that climate worry motivates action) and the anxiety-inhibition hypothesis (that climate anxiety paralyzes action through functional impairment) when applied to these subscales specifically. Several interpretations warrant consideration. The binomial sensitivity analysis, however, suggested a small positive association between cognitive-emotional difficulties and Pro-Environmental Behavior, indicating that climate-related worry may not be behaviorally inert when the outcome’s bounded summed-binary structure is modeled directly.

First, the current sample showed low average scores on both cognitive-emotional difficulties and functional impairment, consistent with a non-clinical, young adult community sample where frank climate anxiety disorder-level symptoms are rare. The limited range on these subscales may have reduced their predictive ability. Second, Mouguiama-Daouda et al. (2022) observed that functional impairment and cognitive-emotional difficulties were linked to depression but not to generalized anxiety, indicating that these subscales might measure a depressive-rumination response to climate change rather than an anxious activation state. Ruminative thinking, unlike worry, has weaker links with motivated behavior change (Nolen-Hoeksema et al., 2008) and may be less likely to predict action.

### Theoretical implications

Our findings suggest that the behavioral engagement subscale of the CAS functions more as an indicator of motivation and identity related to climate change rather than as a symptom of anxiety. This view is consistent with Clayton and Karazsia's (2020) observation that the behavioral engagement items were designed to explore the coexistence of action-oriented responses and anxiety, rather than to be a core element of climate anxiety. Recent validation studies (Mouguiama-Daouda et al., 2022; Wullenkord et al., 2021) have also questioned whether the full four-factor structure of the CAS is empirically supported, often finding that behavioral engagement and personal experience subscales differ from the cognitive-emotional core. These results further support conceptualizing behavioral engagement as a separate motivational dimension, more aligned with PEB research than with psychopathology. Future scale development should consider distinguishing motivation from distress-related constructs when measuring climate change responses in non-clinical populations, specifically young adults.

### Practical and clinical implications

The finding that behavioral engagement, but not distress, predicts pro-environmental action and perceived barriers has practical implications for climate communication and psychological interventions. Environmental communication campaigns that aim to translate concern into action may be more effective when they target motivational and behavioral self-efficacy dimensions rather than emotional arousal per se (Lorenzoni et al., 2007; Steg and Vlek, 2009). Interventions focused on facilitating concrete behavioral commitments, building environmental identity, and reducing structural barriers may better serve both psychological and environmental goals than approaches that amplify anxiety. Conversely, clinical interventions targeting cognitive-emotional difficulties and functional impairment—which were not associated with behavioral outcomes in the present study—may need to be clearly distinguished from behavior-change approaches, recognizing that the relief of climate anxiety distress does not automatically translate into greater environmental engagement (Baudon and Jachens, 2021).

The positive association between behavioral engagement and perceived barriers also suggests a potentially important target for intervention: those most motivated to act may paradoxically face the greatest perceived barriers. Supporting such individuals with information about effective collective action, institutional pathways, and community resources may help translate high motivation into sustained behavior change, preventing the frustration and disengagement that can result from encountering structural barriers without adequate support.

### Robustness to modeling approach

The binomial sensitivity analyses generally reinforced the central conclusion that Behavioral Engagement was the most consistent predictor of climate-relevant outcomes. Its association with both Pro-Environmental Behavior and perceived barriers in the overall models, and with perceived barriers in both sex-stratified models, suggests that this finding is not merely an artifact of the Poisson model specification. However, the sensitivity analyses revealed two model-dependent differences that warrant cautious interpretation. First, Cognitive–Emotional Difficulties emerged as a small positive predictor of Pro-Environmental Behavior in the overall and female-only binomial models, although this association was not observed in the corresponding Poisson models and did not extend to perceived barriers. Given that this finding was not hypothesized, was not consistent across outcomes, and reflected a modest effect, it should be interpreted as preliminary and model-dependent. Second, among males, the positive association between Behavioral Engagement and Pro-Environmental Behavior reached significance in the binomial model, whereas it was only a non-significant trend in the Poisson model. Hence, these sensitivity analyses support the robustness of the Behavioral Engagement findings while indicating that distress-related associations are more sensitive to analytic specification and should be interpreted with caution pending replication.

### Limitations

Several limitations should be noted. First, the sample was restricted to undergraduate students at a single U.S. university and was predominantly White, female, and relatively highly educated, limiting generalizability to more diverse or older populations. The single-site, single-course-pool recruitment strategy, the narrow 18–22 age range, and the demographic skew of the sample together restrict the representativeness of the findings; students recruited from a psychology department research pool at one university are unlikely to be representative of university students more broadly, let alone of young adults outside higher education, and the available documentation does not report the size of the eligible participant pool or the response rate, which further limits our ability to evaluate representativeness or potential non-response or self-selection bias. Relatedly, because the present study is a secondary analysis of an open dataset, the authors had no direct control over the original sampling procedure, recruitment method, measurement design, or data collection process, and several methodological details (e.g., precise recruitment channel, incentive structure) could not be independently verified beyond what is reported in the dataset’s accompanying documentation. Second, the cross-sectional design precludes causal inference; behavioral engagement may both influence and result from PEB, and longitudinal or experimental studies are needed to clarify the direction of causality. Third, both outcomes were measured as simple binary count composites rather than validated psychometric instruments, reducing sensitivity to differences in the frequency and intensity of behaviors and barriers; future research should use validated continuous measures. Fourth, the restricted range of distress-related subscales in this non-clinical sample may have attenuated the associations of these subscales with behavioral outcomes, and replication in samples with higher distress levels is warranted. Fifth, unmeasured variables such as environmental values, self-efficacy, subjective norms, and access to sustainable options may account for shared variance between behavioral engagement and the outcomes. Finally, because all variables were self-reported within the same survey, common method bias cannot be ruled out.

## Conclusion

In the primary Poisson models, the present findings do not indicate that climate anxiety, in general, increases pro-environmental behavior. Rather, they suggest that it is specifically the behavioral and motivational component of the CAS that is linked to climate-related action and awareness of barriers. The present study shows that among the four dimensions of the Climate Anxiety Scale, behavioral engagement significantly predicts both PEB and perceived barriers to climate action in university students. The binomial sensitivity analyses broadly supported the central role of behavioral engagement, while also indicating a limited positive association between cognitive-emotional difficulties and Pro-Environmental Behavior. The distress-related components of climate anxiety—cognitive-emotional difficulties and functional impairment—therefore appear to have more limited and model-dependent behavioral correlates than the motivational dimension of the CAS. These findings underscore the importance of disaggregating dimensions of climate anxiety in research and practice and point to the central role of motivated, action-oriented engagement in climate-relevant behavior. Future work should examine how interventions targeting behavioral engagement and barrier navigation can leverage this motivational orientation to support both psychological well-being and environmental action.

## Data Availability

Publicly available datasets were analyzed in this study. This data can be found at: https://doi.org/10.17632/6bc2mbztmy.1.
