# Supplementary material for: Climate anxiety, pro-environmental behavior, and perceived barriers in university students: analysis of a publicly available dataset from the United States
Source: Front Psychol. 2026 Jul 27;17:1889006. doi: 10.3389/fpsyg.2026.1889006 (PMC13453717; doi:10.3389/fpsyg.2026.1889006)
Supplement: Supplementary file 1 [file Data_sheet_1.pdf]

Supplementary Material

Binomial Sensitivity Analyses for Bounded Summed-Binary Outcomes

Analytic approach. Because each outcome was a sum of five binary indicators, binomial generalized linear models were estimated as sensitivity analyses, treating each outcome as the number of endorsed categories out of five. Odds ratios (ORs) are reported by exponentiating the logit coefficients. CAS subscales were scored using complete item data.

Table S1

Binomial Sensitivity Analysis Predicting Pro-Environmental Behavior

Predictor	B	SE	OR	95% CI	p
Cognitive–Emotional Difficulties	0.040	0.018	1.041	1.005–1.079	.025
Functional Impairment	-0.041	0.031	0.960	0.904–1.019	.179
Personal Experience	0.015	0.017	1.015	0.982–1.050	.370
Behavioral Engagement	0.076	0.012	1.079	1.055–1.104	< .001

Note. Model likelihood-ratio $\chi^2(4) = 102.45$, $p < .001$; $n = 430$. OR = odds ratio. Confidence intervals are exponentiated 95% Wald confidence limits for B.

Table S2

Binomial Sensitivity Analysis Predicting Perceived Barriers

Predictor	B	SE	OR	95% CI	p
Cognitive–Emotional Difficulties	0.027	0.018	1.027	0.991–1.064	.147
Functional Impairment	-0.026	0.031	0.975	0.917–1.036	.409
Personal Experience	0.029	0.017	1.029	0.996–1.065	.089
Behavioral Engagement	0.067	0.011	1.070	1.047–1.093	< .001

Note. Model likelihood-ratio $\chi^2(4) = 95.49$, $p < .001$; $n = 430$. OR = odds ratio. Confidence intervals are exponentiated 95% Wald confidence limits for B.

Table S3

Binomial Sensitivity Analysis Predicting Pro-Environmental Behavior by Sex

Predictor	♀ B	SE	OR	95% CI	p	♂ B	SE	OR	95% CI	p
Cognitive–Emotional Difficulties	0.049	0.020	1.051	1.011–1.092	.012	0.005	0.051	1.005	0.910–1.111	.915
Functional Impairment	-0.022	0.034	0.979	0.916–1.045	.520	-0.078	0.089	0.925	0.778–1.101	.383
Personal Experience	-0.016	0.020	0.984	0.946–1.024	.429	0.106	0.034	1.112	1.040–1.189	.002
Behavioral Engagement	0.078	0.014	1.081	1.053–1.111	< .001	0.053	0.025	1.054	1.004–1.107	.034

Note. Female model (♀): likelihood-ratio $\chi^2(4) = 65.75$, $p < .001$; n = 311. Male model (♂): likelihood-ratio $\chi^2(4) = 31.88$, $p < .001$; n = 118. OR = odds ratio. Confidence intervals are exponentiated 95% Wald confidence limits for B.

Table S4

Binomial Sensitivity Analysis Predicting Perceived Barriers by Sex

Predictor	♀ B	SE	OR	95% CI	p	♂ B	SE	OR	95% CI	p
Cognitive–Emotional Difficulties	0.015	0.020	1.015	0.976–1.055	.452	0.024	0.051	1.024	0.928–1.131	.635
Functional Impairment	-0.023	0.034	0.977	0.914–1.045	.501	0.025	0.087	1.026	0.865–1.217	.771
Personal Experience	0.034	0.020	1.034	0.994–1.076	.095	0.035	0.033	1.035	0.970–1.105	.295
Behavioral Engagement	0.058	0.013	1.060	1.033–1.087	< .001	0.058	0.023	1.059	1.013–1.107	.011

Note. Female model (♀): likelihood-ratio $\chi^2(4) = 48.45$, $p < .001$; n = 311. Male model (♂): likelihood-ratio $\chi^2(4) = 28.90$, $p < .001$; n = 118. OR = odds ratio. Confidence intervals are exponentiated 95% Wald confidence limits for B.
